# On controlled Hamilton and Hamilton–Jacobi differential equations of higher-order

**DOI:** 10.1038/s41598-022-18626-6

**Published:** 2022-08-27

**Authors:** Savin Treanţă, Kamsing Nonlaopon, Muhammad Bilal Khan

**Affiliations:** 1 Department of Applied Mathematics, University Politehnica of Bucharest, 060042 Bucharest, Romania; 2 Academy of Romanian Scientists, 54 Splaiul Independentei, 050094 Bucharest, Romania; 3 Fundamental Sciences Applied in Engineering - Research Center (SFAI), University Politehnica of Bucharest, 060042 Bucharest, Romania; 4 Department of Mathematics, Faculty of Science, Khon Kaen University, Khon Kaen, 40002 Thailand; 5 Department of Mathematics, COMSATS University Islamabad, Islamabad, Pakistan

**Keywords:** Engineering, Mathematics and computing, Physics

## Abstract

In this paper, we investigate the nonlinear dynamics associated with controlled Lagrangians involving higher-order derivatives. More precisely, we establish the controlled higher-order Hamilton ordinary differential equations (ODEs) and Hamilton–Jacobi partial differential equation (PDE) for the considered class of Lagrangians governed by higher-order derivatives of the state variables. Moreover, we formulate and prove an invariance result with respect to the state variable. In addition, in order to validate the theoretical results and to highlight their effectiveness, some illustrative applications are presented.

## Introduction

In this paper, the main goal is to formulate and prove some elements on Hamilton–Jacobi theory governed by single-time controlled higher-order Lagrangians. More precisely, we investigate and establish: controlled Hamilton ODEs, controlled Hamilton–Jacobi PDE, controlled generating function, and controlled canonical momenta. The current paper is based on the first author’s recent papers (see Treanţă^1,2^) and his collaborators (see Treanţă and Udrişte^3^), where only *non-controlled* Lagrangians have been considered. For instance, by considering some multi-time optimization problems, in Treanţă^4^ has established Hamilton-Pfaff PDEs. Also, by using the characteristic system method, some linear higher-order Hamilton–Jacobi PDEs have been studied in Treanţă and Vârsan^5^. Several results on multi-time Hamilton–Jacobi theory, involving Lagrangians of second-order, have been formulated in Treanţă^6^. Moreover, a system of Hamilton–Jacobi PDEs governed by non-controlled Lagrangians of higher-order has been studied in Treanţă^7^.

As we all know, the single-time (classical) Hamilton–Jacobi theory appeared in mechanics out of the desire to characterize the motion of a particle using a wave. Therefore, the Euler–Lagrange and the associated Hamilton ordinary differential equations have been replaced by Hamilton–Jacobi partial differential equations which describe the generating function. With the time, many researchers had a special interest in the study of Hamilton and Hamilton–Jacobi differential equations (see, for instance, see Rochet^8^, Miron^9^, Roman^10^, Krupkova^11^, Motta and Rampazzo^12^, Udrişte, and Ţevy^13^, Cardin and Viterbo^14^, Radjenović et al.^15^, He^16^). Recently, by using the classical Noether’s theorem and a non-standard Legendrian duality, the single-time and multi-time versions of Noether’s result have been investigated for autonomous second-order Lagrangians in Treanţă^17^. Moreover, by using appropriate techniques of calculus of variations and some geometric tools, necessary conditions of optimality have been formulated for the optimization of some simple, multiple or curvilinear integral functionals (governed by Lagrangians of second-order) subject to ordinary/partial differential equation or isoperimetric constraints (see Treanţă^18^). In Krupková and Smetanová^19^ studied the Legendre transformation for regularizable Lagrangians in field theory. Later, Smetanová^20^ stated some results regarding second-order Lagrangians corresponding to 2nd and 3rd order Euler–Lagrange forms. Also, the associated 3rd order Hamiltonian systems have been established. An excellent survey regarding the classical field theory is presented in Giachetta et al.^21^. A theoretical basis for stamp optimization, especially for determining optimal condition for the magnet-controlled transfer printing, is investigated by Linghu et al.^22^. Also, in Pascalis et al.^23^, antiplane wave band gaps are optimized via pre-stress using genetic algorithms. For other different but connected ideas on this topic, the reader is directed to Mahdirajia et al.^24^, Brown and Balakrishnan^25^, and Vlasov^26^ (regarding the kinetic equation with a self-consistent field containing higher-order time derivatives).

Motivated by the ongoing research in this area, in this paper we investigate the nonlinear dynamics associated with controlled Lagrangians involving higher-order derivatives. More precisely, we establish the controlled higher-order Hamilton and Hamilton–Jacobi differential equations for the considered class of Lagrangians governed by higher-order derivatives of the state variables. Moreover, we formulate and prove an invariance result with respect to the state variable.

The paper is structured as follows. “Controlled Hamilton and Hamilton–Jacobi differential equations” section introduces the necessary mathematical tools for establishing the main results of the paper. Theorems 1 and 2 present the controlled partial differential equation of Hamilton–Jacobi type, and an invariance result with respect to the state variable, respectively. These two theorems represent the main results of this paper. Finally, “Conclusions” section concludes the present research work.

## Controlled Hamilton and Hamilton–Jacobi differential equations

This section formulates Hamilton and Hamilton–Jacobi differential equations governed by controlled single-time Lagrangians of higher-order. In this regard, let $k \ge 2$ be a fixed natural number, $\theta \in [\theta _{0},\theta _{1}] \subseteq \mathbb{R},\; s:[\theta _{0},\theta _{1}]\subseteq \mathbb{R} \rightarrow \mathbb{R}^{n},\; s = \left( s^{i}(\theta )\right) ,\; i = \overline{1,n}$, is a $C^{2k}$-class function (called the *state variable*), $s^{(b)}(\theta ) := \frac{d^{b}}{d\theta ^{b}}s(\theta ),\; b \in \{ 1,2,\ldots ,k \}$, and $u:[\theta _{0},\theta _{1}]\subseteq \mathbb{R} \rightarrow \mathbb{R}^{m},\; u = \left( u^{\alpha }(\theta )\right) ,\; \alpha = \overline{1,m}$, is a $C^{1}$-class function (called the *control variable*). The real-valued function

$$\begin{aligned} \mathscr{L}\left( \theta , s(\theta ), s^{(1)}(\theta ),\ldots , s^{(k)}(\theta ), u(\theta )\right) \end{aligned}$$

of $C^{k+1}$-class, named *controlled single-time Lagrangian of higher-order*, depends by $(k+1)n +m+1$ variables. By denoting

$$\begin{aligned} \frac{\partial \mathscr{L}}{\partial s^{(b)i}}\left( \theta ,s(\theta ),s^{(1)}(\theta ),\ldots ,s^{(k)}(\theta ), u(\theta )\right) = p_{bi}(\theta ), \quad b \in \left\{1,2,\ldots ,k \right\}, \end{aligned}$$

and considering the Legendre Transform for the above systems, the relation $\mathscr{L} = s^{(b)i}p_{bi} - \mathscr{H}$ (with Einstein summation) modifies the following controlled simple integral functional

1 $$\begin{aligned} I(s(\cdot ), u(\cdot ))= \int _{\theta _{0}}^{\theta _{1}}\mathscr{L}\left( \theta ,s(\theta ),s^{(1)}(\theta ),\ldots ,s^{(k)}(\theta ), u(\theta )\right) d\theta \end{aligned}$$

into

2 $$\begin{aligned}&J(s(\cdot ), u(\cdot ), p_{1}(\cdot ),\ldots ,p_{k}(\cdot )) \\ &\quad = \int _{\theta _{0}}^{\theta _{1}}\left( s^{(b)i}(\theta )p_{bi}(\theta ) - \mathscr{H}(\theta , s(\theta ), u(\theta ), p_{1}(\theta ),\ldots ,p_{k}(\theta ))\right) d\theta \end{aligned}$$

and the Euler–Lagrange ordinary differential equations of higher-order,

$$\begin{aligned}&\frac{\partial \mathscr{L}}{\partial s^{i}} - \frac{d}{d\theta } \frac{\partial \mathscr{L}}{\partial s^{(1)i}} + \frac{d^{2}}{d\theta ^{2}} \frac{\partial \mathscr{L}}{\partial s^{(2)i}} - \cdots + (-1)^{k}\frac{d^{k}}{d\theta ^{k}} \frac{\partial \mathscr{L}}{\partial s^{(k)i}} = 0, \quad i \in \{ 1,2,\ldots ,n \},\\ &\frac{\partial \mathscr{L}}{\partial u^{\alpha }} - \frac{d}{d\theta } \frac{\partial \mathscr{L}}{\partial u^{(1)\alpha }} + \frac{d^{2}}{d\theta ^{2}} \frac{\partial \mathscr{L}}{\partial u^{(2)\alpha }} - \cdots + (-1)^{k}\frac{d^{k}}{d\theta ^{k}} \frac{\partial \mathscr{L}}{\partial u^{(k)\alpha }} = 0, \quad \alpha \in \{ 1,2,\ldots ,m \}, \end{aligned}$$

associated with (2), are just the *Hamilton ordinary differential equations of higher-order*,

$$\begin{aligned}&\sum _{b = 1}^{k}(-1)^{b+1}\frac{d^{b}}{d\theta ^{b}}p_{bi} = -\frac{\partial \mathscr{H}}{\partial s^{i}}, \quad \frac{d^{b}}{d\theta ^{b}}s^{i} = \frac{\partial \mathscr{H}}{\partial p_{bi}}, \quad b \in \{ 1,2,\ldots ,k \},\\&\frac{\partial \mathscr{H}}{\partial u^{\alpha }} = 0, \quad \alpha \in \{1,2,\ldots ,m \}. \end{aligned}$$

In the following, we shall introduce the Hamilton–Jacobi partial differential equation based on controlled single-time Lagrangians of higher-order.

Let us consider the real-valued function $\mathscr{S}:\mathbb{R} \times \mathbb{R}^{kn} \times \mathbb{R}^{m} \rightarrow \mathbb{R}$ and the controlled constant level sets

$$\begin{aligned} \Sigma _{c}:\mathscr{S}\left( \theta , s, s^{(1)},\ldots ,s^{(k-1)},u \right) = c, \end{aligned}$$

$k \ge 2$ a fixed natural number, where $s^{(b)}(\theta ) := \frac{d^{b}}{d\theta ^{b}}s(\theta ), \; b = \overline{1,k-1}$. Further, we consider that these sets represent some hypersurfaces in $\mathbb{R}^{kn+m+1}$. This means that the normal vector field

$$\begin{aligned} \left( \frac{\partial \mathscr{S}}{\partial \theta }, \frac{\partial \mathscr{S}}{\partial s^{i}}, \frac{\partial \mathscr{S}}{\partial s^{(1)i}},\ldots , \frac{\partial \mathscr{S}}{\partial s^{(k-1)i}},\frac{\partial \mathscr{S}}{\partial u^{\alpha }}, \right) \end{aligned}$$

has linearly independent components. Also, let

$$\begin{aligned} \tilde{\Gamma }:\left( \theta , s^{i}(\theta ), s^{(1)i}(\theta ),\ldots ,s^{(k-1)i}(\theta ),u^{\alpha }(\theta ) \right) ,\quad \theta \in \mathbb{R}, \end{aligned}$$

be a controlled transversal curve associated with the hypersurfaces $\Sigma _{c}$. Then, the function

$$\begin{aligned} c(\theta ) = \mathscr{S}\left( \theta , s(\theta ), s^{(1)}(\theta ),\ldots ,s^{(k-1)}(\theta ),u(\theta ) \right) \end{aligned}$$

has nonzero the total derivative

3 $$\begin{aligned} \frac{dc}{d\theta }(\theta )&= \frac{\partial \mathscr{S}}{\partial \theta }\left( \theta , s(\theta ), s^{(1)}(\theta ),\ldots ,s^{(k-1)}(\theta ),u(\theta )\right) \\ &\quad+ \frac{\partial \mathscr{S}}{\partial s^{i}}\left( \theta , s(\theta ), s^{(1)}(\theta ),\ldots ,s^{(k-1)}(\theta ),u(\theta )\right) s^{(1)i}(\theta ) \\ &\quad+ \sum _{r=1}^{k-1}\frac{\partial \mathscr{S}}{\partial s^{(r)i}}\left( \theta , s(\theta ), s^{(1)}(\theta ),\ldots ,s^{(k-1)}(\theta ),u(\theta )\right) s^{(r+1)i}(\theta ) \\ &\quad+ \frac{\partial \mathscr{S}}{\partial u^{\alpha }}\left( \theta , s(\theta ), s^{(1)}(\theta ),\ldots ,s^{(k-1)}(\theta ),u(\theta )\right) u^{(1)\alpha }(\theta ) \\ &:= \mathscr{L}\left( \theta , s(\theta ), s^{(1)}(\theta ),\ldots ,s^{(k)}(\theta ),u(\theta ),u^{(1)}(\theta )\right) . \end{aligned}$$

Further, by direct computation, it results the *controlled canonical momenta*

$$\begin{aligned} p_{bi}(\theta )&:= \frac{\partial \mathscr{L}}{\partial s^{(b)i}}\left( \theta ,s(\theta ),s^{(1)}(\theta ),\ldots ,s^{(k)}(\theta ),u(\theta ),u^{(1)}(\theta )\right) \\ &= \frac{\partial \mathscr{S}}{\partial s^{(b-1)i}}\left( \theta ,s(\theta ),s^{(1)}(\theta ),\ldots ,s^{(k-1)}(\theta ),u(\theta )\right) , \quad b \in \{1,2,\ldots ,k \} . \end{aligned}$$

### **Definition 1**

The controlled Lagrangian of higher-order

$$\begin{aligned} \mathscr{L}\left( \theta , s(\theta ), s^{(1)}(\theta ),\ldots ,s^{(k)}(\theta ), u(\theta ),u^{(1)}(\theta )\right) \end{aligned}$$

is named super-regular if

$$\begin{aligned} \frac{\partial \mathscr{L}}{\partial s^{(b)i}}\left( \theta ,s(\theta ),s^{(1)}(\theta ),\ldots ,s^{(k)}(\theta ), u(\theta ),u^{(1)}(\theta )\right) = p_{bi}(\theta ), \quad b \in \{1,2,\ldots ,k \} \end{aligned}$$

defines the function of components

$$\begin{aligned} s^{(b)} = s^{(b)}\left( \theta ,s,\ldots , s^{(b-1)},p_{1},\ldots ,p_{k}\right) , \quad b \in \{1,2,\ldots ,k \}. \end{aligned}$$

In these hypotheses, the relations

$$\begin{aligned} s^{(b)} = s^{(b)}\left( \theta ,s,\ldots , s^{(b-1)},u,p_{1},\ldots ,p_{k}\right) ,\quad b \in \{1,2,\ldots ,k \}, \end{aligned}$$

in accordance with Legendre Transform (see Definition 1), can be formulated as

$$\begin{aligned} s^{(b)} = s^{(b)}\left( \theta ,s,\ldots , s^{(b-1)}, u,\frac{\partial \mathscr{S}}{\partial s},\ldots ,\frac{\partial \mathscr{S}}{\partial s^{(k-1)}} \right) ,\quad b \in \{1,2,\ldots ,k \} \end{aligned}$$

and the relation (3) can be rewritten as

4 $$\begin{aligned}&- \frac{\partial \mathscr{S}}{\partial \theta }\left( \theta , s(\theta ), s^{(1)}(\theta ),\ldots ,s^{(k-1)}(\theta ), u(\theta )\right) \\ &\quad = \frac{\partial \mathscr{S}}{\partial s^{i}}\left( \theta , s(\theta ), s^{(1)}(\theta ),\ldots ,s^{(k-1)}(\theta ), u(\theta )\right) \\ &\qquad \cdot s^{(1)i}\left( \theta ,s^{i},u,\frac{\partial \mathscr{S}}{\partial s^{i}}(\cdot ),\ldots ,\frac{\partial \mathscr{S}}{\partial s^{(k-1)i}}(\cdot ) \right) \\ &\qquad + \; \sum _{r=1}^{k-1}\frac{\partial \mathscr{S}}{\partial s^{(r)i}}\left( \theta , s(\theta ), s^{(1)}(\theta ),\ldots ,s^{(k-1)}(\theta ), u(\theta )\right) \\ &\qquad \cdot s^{(r+1)i}\left( \theta ,s^{i},\ldots , s^{(r)i},u,\frac{\partial \mathscr{S}}{\partial s^{i}}(\cdot ),\ldots ,\frac{\partial \mathscr{S}}{\partial s^{(k-1)i}}(\cdot )\right) \\ &\qquad + \frac{\partial \mathscr{S}}{\partial u^{\alpha }}\left( \theta , s(\theta ), s^{(1)}(\theta ),\ldots ,s^{(k-1)}(\theta ), u(\theta )\right) u^{(1)\alpha }\left( \theta \right) \\ &\qquad - \; \mathscr{L}\left( \theta , s(\theta ), s^{(1)}(\theta ),\ldots ,s^{(k)}(\theta ), u(\theta ),u^{(1)}(\theta )\right) . \end{aligned}$$

The duality between the super-regular controlled Lagrangian of higher-order $\mathscr{L}$ and the following controlled Hamilton function gives

$$\begin{aligned}&\mathscr{H}\left( \theta ,s,\ldots ,s^{(k-1)},u,p_{1},\ldots ,p_{k}\right) \\&\quad := s^{(b)i}\left( \theta ,s,\ldots ,s^{(b-1)},u,p_{1},\ldots ,p_{k}\right) \\&\qquad \cdot \frac{\partial \mathscr{L}}{\partial s^{(b)i}}\left( \theta ,s,\ldots ,s^{(k)}(\theta ,s,\ldots ,s^{(k-1)},u, p_{1},\ldots ,p_{k}),u,u^{(1)}\right) \\&\qquad - \; \mathscr{L}\left( \theta ,s,s^{(1)}(\theta ,s,u,p_{1},\ldots ,p_{k}),\ldots ,s^{(k)}(\theta ,s,\ldots s^{(k-1)},u,p_{1},\ldots ,p_{k}),u,u^{(1)}\right) , \end{aligned}$$

(*controlled non-standard Legendre duality of higher-order*) or, for short,

$$\begin{aligned} \mathscr{H} = s^{(b)i}p_{bi} - \mathscr{L}. \end{aligned}$$

Now, by considering all the previous reasoning, we can rewrite (4) as *Hamilton–Jacobi partial differential equation based on controlled Lagrangians of higher-order*,

$$\begin{aligned} (H-J-hig.)\qquad \frac{\partial \mathscr{S}}{\partial \theta } + \mathscr{H}\left( \theta ,s,\ldots ,s^{(k-1)},u,\frac{\partial \mathscr{S}}{\partial s},\frac{\partial \mathscr{S}}{\partial s^{(1)}},\ldots ,\frac{\partial \mathscr{S}}{\partial s^{(k-1)}}\right) = 0. \end{aligned}$$

### *Remark 1*

The above controlled partial differential equation of Hamilton–Jacobi type, based on Lagrangians of higher-order, is equipped with the initial condition

$$\begin{aligned} \mathscr{S}\left( 0, s, s^{(1)},\ldots ,s^{(k-1)}, u \right) = \mathscr{S}_{0}\left( s, s^{(1)},\ldots ,s^{(k-1)}, u \right) , \end{aligned}$$

and the corresponding solution $\mathscr{S}\left( \theta , s, s^{(1)},\ldots ,s^{(k-1)},u \right)$ is named the *controlled generating function* associated with the canonical momenta.

### *Remark 2*

Conversely, let us consider $\mathscr{S}\left( \theta , s, s^{(1)},\ldots ,s^{(k-1)},u \right)$ is a solution of the controlled Hamilton–Jacobi partial differential equation based on Lagrangians of higher-order. Also, we define

$$\begin{aligned} p_{bi}(\theta ) = \frac{\partial \mathscr{S}}{\partial s^{(b-1)i}}\left( \theta ,s(\theta ),s^{(1)}(\theta ),\ldots ,s^{(k-1)}(\theta ), u(\theta )\right) ,\quad b \in \{1,2,\ldots ,k \}. \end{aligned}$$

Taking into acount the above mathematical tools, the following relation is true

$$\begin{aligned}&\int _{\theta _{0}}^{\theta _{1}} \mathscr{L}\left( \theta , s(\theta ), s^{(1)}(\theta ),\ldots ,s^{(k)}(\theta ),u(\theta ), u^{(1)}(\theta )\right) d\theta \\ &\quad = \int _{\theta _{0}}^{\theta _{1}}\left[ s^{(b)i}(\theta )p_{bi}(\theta ) - \mathscr{H}\left( \theta ,s(\theta ),\ldots ,s^{(k-1)}(\theta ),u(\theta ),\right. \right. \\&\qquad \left. \left. \frac{\partial \mathscr{S}}{\partial s}(\cdot ),\frac{\partial \mathscr{S}}{\partial s^{(1)}}(\cdot ),\ldots ,\frac{\partial \mathscr{S}}{\partial s^{(k-1)}}(\cdot ) \right) \right] d\theta \\ &\quad = \int _{\Gamma }\frac{\partial \mathscr{S}}{\partial s^{(b -1)i}}ds^{(b-1)i} + \frac{\partial \mathscr{S}}{\partial \theta } d\theta = \int _{\Gamma }dS, \end{aligned}$$

showing that the cost simple integral functional can be formulated as a curvilinear integral functional which does not depend on the path.

The next theorem represents the first main result derived in the present paper. Its proof is provided by all the above computations and hypotheses.

### **Theorem 1**

*The controlled generating function of the canonical momenta is solution of the Cauchy problem*

$$\begin{aligned}&\frac{\partial \mathscr{S}}{\partial \theta } + \mathscr{H}\left( \theta ,s,\ldots ,s^{(k-1)},u,\frac{\partial \mathscr{S}}{\partial s},\frac{\partial \mathscr{S}}{\partial s^{(1)}},\ldots ,\frac{\partial \mathscr{S}}{\partial s^{(k-1)}}\right) = 0\\&\mathscr{S}\left( 0, s, s^{(1)},\ldots ,s^{(k-1)},u \right) = \mathscr{S}_{0}\left( s, s^{(1)},\ldots ,s^{(k-1)},u\right) = \text {given}. \end{aligned}$$

### *Example 1*

Let $\theta$ be the time, $u=(u^\alpha )$ is the control vector, and $s=(s^i)$ is the vector of spatial coordinates. Consider the function (operator) $H_1=I$ is associated with the information as a measure of organization (synergy and purpose), the function (operator) $H_2=H$ is associated with the energy as a measure of movement, the function $S^1$ is the generating function for entropy, and the function $S^2$ is the generating function for action. A controlled system of partial differential equations having the following form

$$\begin{aligned}&\frac{\partial S^1}{\partial \theta } + H_1\left( \theta ,s, u,\frac{\partial S^1}{\partial s}, \frac{\partial S^2}{\partial s}\right) = 0,\\ &\frac{\partial S^2}{\partial \theta } + H_2\left( \theta ,s,u,\frac{\partial S^1}{\partial s}, \frac{\partial S^2}{\partial s}\right) = 0 \end{aligned}$$

is called *physical control*. This kind of system can be written by using the real vector function $S= (S^1,S^2):\mathbb{R} \times \mathbb{R}^{n}\times \mathbb{R}^{m} \rightarrow \mathbb{R}$.

The following theorem formulates the second main result of this paper. It establishes, under some hypotheses, the invariance with respect to the state variable *s* of

$$\begin{aligned} \frac{dS}{d\theta } + \mathscr{H}\left( \theta ,s,\ldots ,s^{(k-1)},u,\frac{\partial \mathscr{S}}{\partial s},\frac{\partial \mathscr{S}}{\partial s^{(1)}},\ldots ,\frac{\partial \mathscr{S}}{\partial s^{(k-1)}}\right) . \end{aligned}$$

### **Theorem 2**

If the equality

$$\begin{aligned}&\mathscr{L}\left( \theta , s(\theta ), s^{(1)}(\theta ),\ldots ,s^{(k)}(\theta ),u(\theta ), u^{(1)}(\theta ) \right) \\&\quad = \frac{\partial \mathscr{S}}{\partial \theta }\left( \theta , s(\theta ), s^{(1)}(\theta ),\ldots ,s^{(k-1)}(\theta ) ,u(\theta )\right) \\&\qquad + \; \frac{\partial \mathscr{S}}{\partial s^{i}}\left( \theta , s(\theta ), s^{(1)}(\theta ),\ldots ,s^{(k-1)}(\theta ),u(\theta ) \right) s^{(1)i}(\theta ) \\&\qquad + \; \sum _{r = 1}^{k-1}\frac{\partial \mathscr{S}}{\partial s^{(r)i}}\left( \theta , s(\theta ), s^{(1)}(\theta ),\ldots ,s^{(k-1)}(\theta ) ,u(\theta )\right) s^{(r+1)i}(\theta ) \\&\qquad + \; \frac{\partial \mathscr{S}}{\partial u^{\alpha }}\left( \theta , s(\theta ), s^{(1)}(\theta ),\ldots ,s^{(k-1)}(\theta ),u(\theta ) \right) u^{(1)\alpha }(\theta ) \end{aligned}$$

is fulfilled and the associated domain is convex, then

$$\begin{aligned} \frac{dS}{d\theta } + \mathscr{H}\left( \theta ,s,\ldots ,s^{(k-1)},u,\frac{\partial \mathscr{S}}{\partial s},\frac{\partial \mathscr{S}}{\partial s^{(1)}},\ldots ,\frac{\partial \mathscr{S}}{\partial s^{(k-1)}}\right) \end{aligned}$$

is invariant with respect to *s*.

### *Proof*

By computation, we obtain

$$\begin{aligned}&\frac{\partial \mathscr{L}}{\partial s^{j}}\left( \theta , s(\theta ), s^{(1)}(\theta ),\ldots ,s^{(k)}(\theta ),u(\theta ),u^{1}(\theta ) \right) \\&\quad = \frac{\partial ^{2} \mathscr{S}}{\partial \theta \partial s^{j}}\left( \theta , s(\theta ), s^{(1)}(\theta ),\ldots ,s^{(k-1)}(\theta ),u(\theta ) \right) \\ &\qquad + \frac{\partial ^{2} \mathscr{S}}{\partial s^{i} \partial s^{j}}\left( \theta , s(\theta ), s^{(1)}(\theta ),\ldots ,s^{(k-1)}(\theta ) ,u(\theta )\right) s^{(1)i}(\theta ) \\&\qquad + \sum _{r = 1}^{k-1}\frac{\partial ^{2} \mathscr{S}}{\partial s^{(r)i} \partial s^{j}}\left( \theta , s(\theta ), s^{(1)}(\theta ),\ldots ,s^{(k-1)}(\theta ),u(\theta ) \right) s^{(r+1)i}(\theta ), \\ &\qquad + \frac{\partial ^{2} \mathscr{S}}{\partial u^{\alpha } \partial s^{j}}\left( \theta , s(\theta ), s^{(1)}(\theta ),\ldots ,s^{(k-1)}(\theta ) ,u(\theta )\right) u^{(1)\alpha }(\theta ) \end{aligned}$$

equivalent with

$$\begin{aligned}&- \frac{\partial \mathscr{H}}{\partial s^{j}} \left( \theta ,s(\theta ),\ldots ,s^{(k-1)}(\theta ),u(\theta ),\frac{\partial \mathscr{S}}{\partial s}(\cdot ),\frac{\partial \mathscr{S}}{\partial s^{(1)}}(\cdot ),\ldots ,\frac{\partial \mathscr{S}}{\partial s^{(k-1)}}(\cdot ) \right) \\ &\quad = \frac{\partial ^{2} \mathscr{S}}{\partial \theta \partial s^{j}}\left( \theta , s(\theta ), s^{(1)}(\theta ),\ldots ,s^{(k-1)}(\theta ),u(\theta ) \right) \\ &\qquad + \frac{\partial ^{2} \mathscr{S}}{\partial s^{i} \partial s^{j}}\left( \theta , s(\theta ), s^{(1)}(\theta ),\ldots ,s^{(k-1)}(\theta ) ,u(\theta )\right) s^{(1)i}(\theta ) \\ &\qquad + \sum _{r = 1}^{k-1}\frac{\partial ^{2} \mathscr{S}}{\partial s^{(r)i} \partial s^{j}}\left( \theta , s(\theta ), s^{(1)}(\theta ),\ldots ,s^{(k-1)}(\theta ),u(\theta ) \right) s^{(r+1)i}(\theta ), \\ &\qquad + \frac{\partial ^{2} \mathscr{S}}{\partial u^{\alpha } \partial s^{j}}\left( \theta , s(\theta ), s^{(1)}(\theta ),\ldots ,s^{(k-1)}(\theta ) ,u(\theta )\right) u^{(1)\alpha }(\theta ) \end{aligned}$$

or,

$$\begin{aligned} \frac{\partial }{\partial s^{j}}\left[ \frac{dS}{d\theta } + \mathscr{H}\left( \theta ,s,\ldots ,s^{(k-1)},u,\frac{\partial \mathscr{S}}{\partial s},\frac{\partial \mathscr{S}}{\partial s^{(1)}},\ldots ,\frac{\partial \mathscr{S}}{\partial s^{(k-1)}}\right) \right] = 0, \end{aligned}$$

involving

$$\begin{aligned}&\frac{dS}{d\theta } + \mathscr{H}\left( \theta ,s,\ldots ,s^{(k-1)},u,\frac{\partial \mathscr{S}}{\partial s},\frac{\partial \mathscr{S}}{\partial s^{(1)}},\ldots ,\frac{\partial \mathscr{S}}{\partial s^{(k-1)}}\right) \\ &\quad =f(\theta ,s^{(1)}(\theta ),\ldots ,s^{(k)}(\theta ),u(\theta ),u^{1}(\theta )) \end{aligned}$$

and the proof is complete. $\square$

### Illustrative applications

 Next, we will formulate and investigate two applications associated with the studied formalism in the paper.

1. Let us extremize the following simple integral functional
  $$\begin{aligned} I\left( s(\cdot ), u(\cdot )\right)&=\int _{\theta _{0}}^{\theta _{1}}\mathscr{L}\left( s(\theta ),\dot{s}(\theta ),\ddot{s}(\theta ),u(\theta ), \theta \right) d\theta \\ &= - \int _{0}^{1}\left( s(\theta )+u^{2}(\theta )\right) d\theta \end{aligned}$$
  subject to the restrictions
  $$\begin{aligned} \dot{s}(\theta ) = u(\theta ), \quad s(0)=0, \quad s(1) = x_{1} = \text {given}. \end{aligned}$$

### Solution

To study the above constrained variational control problem, we consider the following auxiliary Lagrangian

$$\begin{aligned} \mathscr{L}_{1}\left( s(\theta ),\dot{s}(\theta ),\ddot{s}(\theta ),u(\theta ), \theta \right) = -\left( s(\theta )+u^{2}(\theta ) \right) + p(\theta ) \left( u(\theta )-\dot{s}(\theta ) \right) , \end{aligned}$$

that determines the following Euler–Lagrange type equations [or Hamilton type equations, if we consider $\mathscr{H} = s^{(b)i}p_{bi} - \mathscr{L} = \dot{s}(\theta )p(\theta )+ s(\theta )+u^{2}(\theta )$]

$$\begin{aligned} \dot{p}(\theta )&= 1 \Rightarrow p(\theta ) = \theta +c, \quad c \in \mathbb{R},\\ &-2 u(\theta ) + p(\theta ) = 0 \Rightarrow u(\theta ) = \frac{p(\theta )}{2} = \frac{\theta +c}{2}, \end{aligned}$$

and

$$\begin{aligned} u(\theta ) = \dot{s}(\theta ). \end{aligned}$$

By combining the last two relations, we obtain

$$\begin{aligned} s(\theta ) = \frac{\theta ^{2}}{4}+\frac{c \theta }{2} + b, \quad b \in \mathbb{R} \end{aligned}$$

and taking into account the conditions $s(0)=0, \; s(1) = x_{1}$, it follows $b = 0, \; c = 2\left( x_{1}-\frac{1}{2}\right)$.

(2) The following application takes into account the equations of multi-time dynamics generated by suitable Lagrangians. Let us extremize the mechanical work provided by the controlled variable force $\overline{V} = (s^{2}(\theta ) + u^{2}(\theta ),s^{2}(\theta ) + u^{2}(\theta ))$ to move its application point along the following piecewise smooth curve $\Upsilon _{0,1}$, included in $[0,1]^{2}$, joining the points $(0,0), \; (1,1)$, so that $\int _{\Upsilon _{0,1}}s_{\theta ^{1}}(\theta )d\theta ^{1} + s_{\theta ^{2}}(\theta )d\theta ^{2} = 0$ (path-independent curvilinear integral) and the boundary conditions $s(0,0) = 0$, $s(1,1) = 0$ are satisfied.
  *Solution.* We consider the following controlled curvilinear integral functional
  $$\begin{aligned} J\left( s(\cdot ),u(\cdot )\right) = \int _{\Upsilon _{0,1}}\left( s^{2}(\theta ) + u^{2}(\theta )\right) d\theta ^{1}+\left( s^{2}(\theta ) + u^{2}(\theta )\right) d\theta ^{2} \end{aligned}$$
  subject to: $\int _{\Upsilon _{0,1}}s_{\theta ^{1}}(\theta )d\theta ^{1} + s_{\theta ^{2}}(\theta )d\theta ^{2} = 0$ (path-independent curvilinear integral) and the boundary conditions $s(0,0) = 0, \; s(1,1) = 0$. The path-independence associated with the cost functional $J\left( s(\cdot ),u(\cdot )\right)$ gives the relation
  $$\begin{aligned} s\left( \frac{\partial s}{\partial \theta ^{2}}-\frac{\partial s}{\partial \theta ^{1}}\right) = u\left( \frac{\partial u}{\partial \theta ^{1}}-\frac{\partial u}{\partial \theta ^{2}}\right) . \end{aligned}$$
  Also, the corresponding Lagrange 1-form has the components
  $$\begin{aligned}&\mathscr{L}_{11} = s^{2}(\theta ) + u^{2}(\theta ) + p\left( y_{\theta ^{1}}(\theta ) - s_{\theta ^{1}}(\theta )\right) , \\ &\mathscr{L}_{12} = s^{2}(\theta ) + u^{2}(\theta ) + p\left( y_{\theta ^{2}}(\theta ) - s_{\theta ^{2}}(\theta )\right) \end{aligned}$$
  and the extremals are provided by the Euler–Lagrange system of PDEs
  $$\begin{aligned}&2s + \frac{\partial p}{\partial \theta ^{1}} = 0, \quad 2s + \frac{\partial p}{\partial \theta ^{2}} = 0,\\ &2u = 0,\\&y_{\theta ^{1}}(\theta ) - s_{\theta ^{1}}(\theta ) = 0, \quad y_{\theta ^{2}}(\theta ) - s_{\theta ^{2}}(\theta ) = 0, \end{aligned}$$
  implying that $(s^{*},u^{*}) = (0,0)$ is the optimal solution of the considered isoperimetric constrained variational control problem.
  Also, it can be easily verified that Theorem 2 is satisfied for the above two illustrative applications.

## Conclusions

In this paper, we have established the nonlinear dynamics associated with a class of controlled Lagrangians involving higher-order derivatives. Concretely, we have formulated the controlled higher-order Hamilton and Hamilton–Jacobi differential equations for the considered Lagrangians governed by higher-order derivatives of the state variables. Also, we have formulated and proved an invariance result with respect to the the state variable. An illustrative application of the theoretical results obtained in the paper was also provided.

## Data Availability

The datasets used and/or analysed during the current study available from the corresponding author on reasonable request.

## Additional information

Correspondence and requests for materials should be addressed to K.N.
